# The Flora of Healing Wounds

**Published:** 1898-04-02

**Authors:** 


### THE FLORA OF HEALING WOUNDS.

Mr. George Stoker has made a series of observations in regard to the reaction of healing and non-healing ulcers, which are of some interest. It was found that all healing wounds were but slightly alkaline, but that when the healing process was arrested they became highly so, the alkalinity rapidly diminishing again as soon as healing began. This greater alkalinity which occurred when healing was arrested was accompanied by an almost complete disappearance of staphylococci from the wound or ulcer and the appearance of rod bacteria, especially bacillus fluorescens. Perhaps surgeons have been somewhat too ready to assume the sterility of healing ulcers, and clearly a careful study of their flora is very desirable.

